# Genome Sequence of *Streptomyces violaceusniger* Strain SPC6, a Halotolerant Streptomycete That Exhibits Rapid Growth and Development

**DOI:** 10.1128/genomeA.00494-13

**Published:** 2013-07-18

**Authors:** Ximing Chen, Binglin Zhang, Wei Zhang, Xiukun Wu, Manxiao Zhang, Tuo Chen, Guangxiu Liu, Paul Dyson

**Affiliations:** Key Laboratory of Desert and Desertification, Cold and Arid Regions Environmental and Engineering Research Institute, Chinese Academy of Sciences, Lanzhou, Gansu, Chinaa; Institute of Life Science, School of Medicine, Swansea University, Singleton Park, Swansea, United Kingdomb

## Abstract

*Streptomyces violaceusniger* strain SPC6 is a halotolerant streptomycete isolated from the Linze desert in China. The strain has a very high growth rate and a short life cycle for a streptomycete. For surface-grown cultures, the period from spore germination to formation of colonies with mature spore chains is only 2 days at 37°C. Additionally, the strain is remarkably resistant to osmotic, heat, and UV stress compared with other streptomycetes. Analysis of the draft genome sequence indicates that the strain has the smallest reported genome (6.4 Mb) of any streptomycete. The availability of this genome sequence allows us to investigate the genetic basis of adaptation for growth in an extremely arid environment.

## GENOME ANNOUNCEMENT

*Streptomyces violaceusniger* strain SPC6, isolated from the Linze desert in China, is a halotolerant strain that can be grown in medium supplemented with 0 M to 1 M NaCl. This halotolerance is one example of an adaptation for growth in an arid desert environment. In addition, SPC6 has a very high growth rate and short life cycle compared with other streptomycetes, including the model organism *Streptomyces coelicolor*. For surface-grown cultures, the period from spore germination to formation of colonies with mature spore chains is only 2 days at 37°C. Additionally, it can produce antibiotics that inhibit the growth of other bacteria. The availability of this genome sequence will underpin investigations into stress adaptations, cell division, morphological development, and antibiotic production.

The raw sequence was generated using a shotgun approach employing Illumina technology (1). Two Illumina shotgun libraries (insert size of ~500 bp and reads totaling 1,376 Mb, and insert size of ~6 kb and reads totaling 1,193 Mb) were sequenced. Reads were assembled using SOAP*denovo* (2) into 15 scaffolds. Putative protein-coding sequences were predicted using Glimmer3 (3). Functional annotation was based on BLASTp results against the NR, Swiss-Prot, TrEMBL, NR, GO, and COG databases. tRNA and rRNA genes were predicted with tRNAscan (4) and RNAmmer (5), respectively. Secondary metabolic gene clusters were predicted using antiSMASH (6). The draft genome of SPC6 consists of one chromosome of 6,457,341 bp with 73.37% G+C content. Six 23S rRNA operons, 6 5S rRNA operons, 7 16S rRNA operons, 75 tRNA genes, and 5,927 coding sequences (CDSs) were identified in the genome. Over 56.8% (3,368) of the predicted open reading frames (ORFs) present in the SPC6 genome cannot be assigned a putative function. It appears that 20.6% (1,219) of hypothetical proteins are unique to this bacterium, with no matches to any other known sequence.

In relation to stress adaptation, 43 genes were identified as involved in the synthesis of trehalose (7, 8), glycogen, and glycine betaine. Remarkably, there are 106 predicted *proV* genes (l-proline/glycine betaine ABC transport system permease protein), and 4 *proP* genes, 4 *proW* genes, and 1 *proX* gene were assigned in this genome. In addition, the genome encodes 28 extracytoplasmic function (ECF) sigma factors and 17 chaperone proteins that might contribute to its resistance to heat stress. Seventeen gene clusters for secondary metabolite biosynthesis were predicted from the genome: 1 predicted cluster of 8 genes for biosynthesis of ectoine, an osmoprotectant; 2 gene clusters (52 genes) for the synthesis of siderophores, which are involved in obtaining soluble Fe3^+^ from the environment; 3 hybrid type I polyketide synthase (PKS)-nonribosomal peptide synthase (NRPS) clusters (one of which is related to the polyene DKxanthene cluster [9], which is involved in sporulation regulation in *Myxococcus*); 4 NRPS clusters; 1 NRPS-lantibiotic cluster; 1 type III PKS gene cluster; 1 type III PKS-terpene hybrid biosynthesis cluster; 3 clusters for terpene biosynthesis; and 1 cluster related to the biosynthetic gene cluster for the aminoglycoside istamycin.

### Nucleotide sequence accession numbers.

This whole-genome shotgun project has been deposited at DDBJ/EMBL/GenBank under the accession no. ASHX00000000. The version described in this paper is first version, accession no. ASHX01000000.
